# The Milan system for reporting salivary gland cytopathology—A single‐center study of 2156 cases

**DOI:** 10.1002/cam4.5914

**Published:** 2023-04-16

**Authors:** Giulia Tochtermann, Miriam Nowack, Cristina Hagen, Niels J. Rupp, Kristian Ikenberg, Martina A. Broglie, Francesca Saro, Daniela Lenggenhager, Peter K. Bode

**Affiliations:** ^1^ Department of Pathology and Molecular Pathology University Hospital Zurich Zurich Switzerland; ^2^ Faculty of Medicine University of Zurich Zurich Switzerland; ^3^ Department of Otorhinolaryngology, Head and Neck Surgery University Hospital of Zurich Zurich Switzerland; ^4^ Present address: Departement of Pathology and Molecular Pathology University Hospital Zurich, Zurich, Switzerland and Institute of Pathology, Cantonal Hospital St. Gallen Switzerland; ^5^ Present address: Departement of Pathology and Molecular Pathology University Hospital Zurich, Zurich, Switzerland and GZO‐Zurich Regional Health Centre Wetzikon Switzerland; ^6^ Present address: Departement of Pathology and Molecular Pathology University Hospital Zurich, Zurich, Switzerland and Institute of Pathology, Cantonal Hospital Winterthur Switzerland

**Keywords:** cytology, fine‐needle aspiration (FNA), Milan system for reporting salivary gland cytopathology (MSRSGC), risk of malignancy (ROM), salivary gland tumors

## Abstract

**Background:**

Fine‐needle aspiration cytology (FNAC) represents an important diagnostic tool for the workup of salivary gland (SG) lesions. The Milan System for Reporting Salivary Gland Cytopathology (MSRSGC) is a six‐tiered system for standardizing diagnoses and improvement of communication between pathologists and clinicians, providing risk of malignancy (ROM) rates for every category. The aims of the present study were (i) to validate the use of MSRSGC in a large series of SG FNAC in a tertiary center in Switzerland, (ii) to determine ROM for each category and compare them with data from MSRSGC and similar studies, and (iii) to investigate whether there were relevant differences of non‐diagnostic results between fine‐needle aspirations (FNA) performed by cytopathologists compared to non‐cytopathologists.

**Methods:**

The files of the department of Pathology in the University Hospital Zurich (UHZ) were searched for SG FNAC between 2010 and 2019. The MSRSGC guidelines were applied retrospectively. Furthermore, ROM, risk of neoplasia (RON), sensitivity, and specificity were calculated based on the cases with histopathological follow‐up.

**Results:**

A total of 2156 SG FNAC including 753 cases with histopathological follow‐up were evaluated. Generally, ROM was within the range of values provided by MSRSGC, with some minor deviations. Sensitivity was 94.6%, and specificity was 99.3%.

**Conclusions:**

Our study confirms the usefulness of MSRSGC. In addition, it provides a detailed insight into the wide spectrum of SG FNAC. Finally, we showed that the rate of non‐diagnostic FNA was significantly lower in FNAs performed by cytopathologists compared to non‐cytopathologists.

## Graphical abstract

This study validated the use of The Milan System for Reporting Salivary Gland Cytopathology in a large series of 2156 cases in a tertiary center in Switzerland. ROM was determined from 753 cases with histopathological follow‐up and compared with data from MSRSGC and other studies.

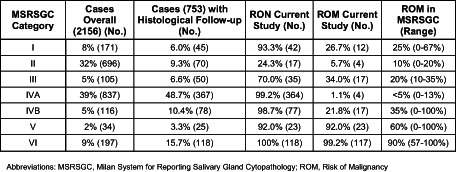

## 1 INTRODUCTION

Salivary gland (SG) tumors are rare, representing about 3% of all tumors in the head and neck region worldwide.^1^, ^2^ Since the beginning of routine application of fine‐needle aspiration cytology (FNAC) in Sweden at the Karolinska institute in 1964, it has gained importance in the diagnostic workup of SG lesions and is here to stay.^3^ The popularity of FNAC as a preoperative assessment tool for SG lesions is based not only on the good accessibility of these lesions, the general simplicity of the procedure and its minimal invasiveness, but also on the favorable cost–benefit ratio, low complication rate, as well as high sensitivity (83%–92%) and specificity (93%–100%).^4^, ^5^, ^6^, ^7^ Besides the aforementioned rarity of SG tumors, their wide range of subtypes, their overlapping morphological features, and heterogeneity results in an intermediate accuracy rate (60%–75%), when a specific diagnosis is rendered.^5^, ^8^ Only in a minority of benign neoplasms (namely classic pleomorphic adenoma and Warthin tumor), FNAC can provide a specific diagnosis^9^ with high diagnostic accuracy rate (>80%).^10^, ^11^, ^12^

In 2018, an international group of experts supported by the American Society of Cytopathology and the International Academy of Cytology developed a classification system called The Milan System for Reporting Salivary Gland Cytopathology (MSRSGC).^9^, ^13^ The goal was to provide an evidence‐based reporting system, to standardize diagnoses, to highlight the essential information for clinicians, and to improve communication between pathologists and clinicians. Thus, the expert group developed a six‐tiered diagnostic scheme. For each group, the risk of malignancy (ROM) was reported and a recommendation for further clinical management was made.^13^ Since the publication of the MSRSGC in 2018, its applicability has been appraised in several studies, partially with low case numbers.^5^, ^6^, ^9^, ^14^, ^15^, ^16^, ^17^, ^18^, ^19^, ^20^, ^21^, ^22^, ^23^, ^24^, ^25^, ^26^, ^27^

The aim of our retrospective, single‐center study was to validate the use of MSRSGC in a large series of FNAC of SG lesions and to determine ROM and risk of neoplasia (RON) for each category. In addition, we investigated whether there were statistically relevant differences in the number of non‐diagnostic results between FNA performed by cytopathologists and non‐cytopathologists.

## 2 MATERIALS AND METHODS

The department of Pathology and Molecular Pathology at the University Hospital Zurich (UHZ) Switzerland operates a walk‐in clinic by cytopathologists with specialized training in ultrasound‐guided FNA. On average, >1500 FNA are performed annually, with most lesions occurring in the head and neck region. A retrospective search of the UHZ pathology database was done for SG FNAC cases in the period between 2010 and 2019. Inclusion criteria were intraglandular lesions of any SG, including metastases and lymphomas of intraglandular lymph nodes. Lesions located near, but outside a SG were excluded. Since some patients had multiple SG lesions, a distinction was made between the number of patients and the number of lesions in our study. Repeat FNA of the same lesion were not considered as a separate case number. Tumor size and location, as well as demographic data such as gender and age of patients were recorded. Furthermore, a distinction was made between FNA performed by a cytopathologist and FNA performed by non‐cytopathologists. A cell block was prepared for each of the internally performed FNA, which was not the case for FNA sent by external physicians. Immunohistochemistry was not used as standard for the diagnoses; this was decided individually on a case‐by‐case basis.

The MSRSGC guidelines were assigned retrospectively by two cytopathologists (G.T. and M.N.). In cases of discrepancy, consensus was found with a third cytopathologist (P.B.). For the calculation of ROM and RON of each category, only cases with surgical follow‐up and definite histopathological diagnosis were included. In case of repeat FNA, only the Milan category of the first FNA was considered for the calculation of ROM and RON. ROM represents the ratio between the number of FNA and the number of malignancies in the definite histology. RON represents the ratio between the number of FNA and the number of neoplasms (benign and malignant). Furthermore, sensitivity and specificity as well as positive (PPV) and negative predictive value (NPV) were determined. For statistical analysis of sensitivity and specificity to predict malignancy, we grouped categories V and VI as positive and II and IVA as negative. For predicting neoplasm, we grouped categories IVA, IVB, V, and VI as positive and II as negative.

## 3 RESULTS

In the period between 2010 and 2019, a total of 2256 FNAC of SG lesions in 2101 patients were evaluated at the UHZ. Out of these, 100 cases were repeat FNA, resulting in 2156 cases that were retrospectively classified according to the MSRSGC. The cohort consisted of 55% men and 45% women with a mean age of 58.3 years. Most lesions were located in the parotid gland (82.7%); 14.7% occurred in the submandibular and 0.6% in the sublingual glands. The average size was 20.2 mm. It did not significantly differ between localizations, but the lesions were smaller in MSRSGC category I and II (18.5 and 16.6 mm) compared to categories III‐VI (22.8, 21.7, 22.3, 24.3, and 22.1 mm). 87.6% of all FNA were performed by cytopathologists in the walk‐in clinic, whereas 12.4% were conducted by clinicians and sent for diagnosis. Clinical and pathological data are summarized in Table 1.

**TABLE 1:** Patient characteristics and overview over details of FNAC.

Variable	*N*. (%)
Patients	2101
Sex	
Male	1156 (55)
Female	945 (45)
Age (years), mean ± SD	58.3 ± 17.1
Salivary gland FNAC overall	2156
Location	
Parotid gland	1784 (82.7)
Submandibular gland	317 (14.7)
Sublingual gland	12 (0.6)
Salivary gland, unknown location	43 (2.0)
Average size of lesions	20.2 mm
FNAC performance	
In‐house, by cytopathologists	1889 (87.6)
By external physicians	267 (12.4)
Salivary gland FNAC with histopathologic diagnosis	753 (34.9)
Histopathologic diagnosis category	
Non‐neoplastic lesion	77 (10.2)
Benign neoplasm	482 (64.0)
Malignant neoplasm	194 (25.8)

Abbreviations: FNAC, fine‐needle aspiration cytology; SD, standard deviation.

In 95.5%, only one FNA was performed. A second FNA was performed in 4.3% and a third FNA in 0.2%. Repeat FNA occurred most frequently in lesions interpreted as category I (41%), followed by category II (22%), IVA (16%), and III (14%). Repeat FNA delivered a diagnostic result in 87%—mostly a category IVA (38%), followed by category II (24%), VI (12%), III (6%), and IVB (5%), whereas 13% were still non‐diagnostic.

**FIGURE 1:**
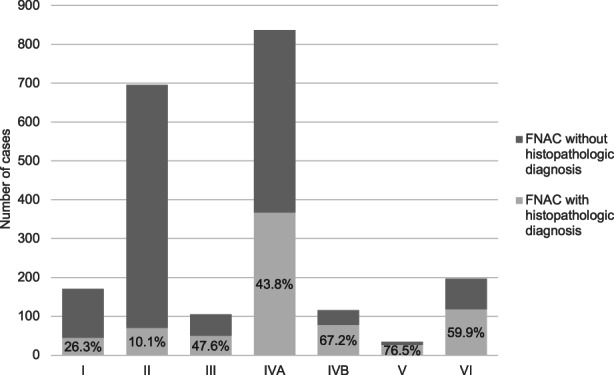
FNAC with histopathological follow‐up in the different MSRSGC categories.

Histological workup was available in 753 cases, revealing benign neoplasms in 64%, malignant neoplasms in 25.8%, and non‐neoplastic lesions in 10.2% (Table 1). The most frequent preoperative FNAC result was benign SG neoplasm (48.7%), followed by malignant SG neoplasms (15.7%). Histological correlation was mainly possible in MSRSGC category V (76.5%), IVB (67.2%), and VI (59.9%), whereas surgery was performed less often in category III (47.6%), followed by category I (26.3%) and category II (10.1%) (Figure 1).

Of the 753 cases with histologic follow‐up, pleomorphic adenoma (33%) and Warthin tumor (25%) were the most frequent benign neoplasms, followed by lipoma (1.9%) and basal cell adenoma (1.7%). The most frequent malignant neoplasm was lymphoma (5.7%) followed by metastatic squamous cell carcinoma (3.9%) and melanoma (2.5%). The most common primary SG carcinomas were mucoepidermoid carcinoma (1.9%), salivary duct carcinoma (1.9%), acinic cell carcinoma, and adenoid cystic carcinoma (each 1.7%). The most frequent non‐neoplastic lesions were cysts (3.6%) followed by inflammatory processes (2.9%). The detailed data are provided in a table in Table S1.

Sensitivity and specificity of FNAC for determining neoplastic versus non‐neoplastic SG processes were 97.2% and 89.8%, respectively, with a PPV of 99.0% and a NPV of 75.7%. Sensitivity and specificity for predicting malignancy by MSRSGC were 94.6% and 99.3%, respectively, with a PPV of 97.9% and a NPV of 98.2%. ROM and RON are summarized in Table 2. We compared our ROM with several previously conducted studies that also assessed the reliability of the MSRSGC. Only studies that examined at least 200 cases with histopathologic correlation were included. Details are summarized in Table 3.

**TABLE 2:** MSRSGC category of cases overall and cases only with histopathologic diagnosis and appurtenant ROM and RON.

MSRSGC category	Cases overall (2156) (no.)	Cases (753) with histological follow‐up (no.)	RON current study (no.)	ROM current study (no.)	ROM in MSRSGC (range)
I	8% (171)	6.0% (45)	93.3% (42)	26.7% (12)	25% (0%–67%)
II	32% (696)	9.3% (70)	24.3% (17)	5.7% (4)	10% (0%–20%)
III	5% (105)	6.6% (50)	70.0% (35)	34.0% (17)	20% (10%–35%)
IVA	39% (837)	48.7% (367)	99.2% (364)	1.1% (4)	<5% (0%–13%)
IVB	5% (116)	10.4% (78)	98.7% (77)	21.8% (17)	35% (0%–100%)
V	2% (34)	3.3% (25)	92.0% (23)	92.0% (23)	60% (0%–100%)
VI	9% (197)	15.7% (118)	100% (118)	99.2% (117)	90% (57%–100%)

Abbreviations: MSRSGC, Milan System for Reporting Salivary Gland Cytopathology; ROM, risk of malignancy.

**TABLE 3:** Comparison of ROM for the MSRSGC categories with other studies with histopathologic follow‐up published 2018–2022 with over 200 cases.

Study	FNAC^a^ No.	MSRSGC category							Time period (years)	Included localizations
		I (%)	II (%)	III (%)	IVA (%)	IVB (%)	V (%)	VI (%)		
Thiryayi 2018^23^—unicentric	283 (287)	8.5	1.6	0.0	1.9	26.7	100.0	100.0	3	Parotid & submandibular gland, neck NOS, neck level Ib and II
Viswanathan 2018^15^—unicentric	373 (627)	6.7	7.1	38.9	5.0	34.2	92.9	92.3	5	Parotid & submandibular gland, minor salivary glands
Chen 2019^20^—unicentric	349 (1020)	8.6	15.4	36.8	2.6	32.3	71.4	100.0	10	Parotid & submandibular gland, salivary gland NOS
Mazzola 2019^16^—unicentric	366 (375)	19.0	11.8	25.0	5.5	50.0	71.4	94.6	8	Parotid & submandibular & sublingual gland, minor salivary glands
Choy 2019^19^—unicentric	376 (367)	14.5	26.7	29.3	2.7	19.1	87.5	100.0	14	Parotid gland
Dubucs 2019^6^—unicentric	216 (328)	34.0	0.0	0.0	3.1	45.5	68.8	100.0	4	Parotid & submandibular gland, minor salivary glands
Maleki 2019^24^—multicentric	333 (734)	10.6	7.5	27.6	3.2	41.9	82.3	93.6	5	Submandibular gland
Savant 2019^5^—unicentric	199 (331)	0.0	0.0	33.3	0.8	40.9	100.0	100.0	6	Parotid & submandibular gland, minor salivary glands, salivary gland NOS^b^
Song 2019^18^—unicentric	429 (893)	17.8	14.3	30.6	2.2	46.6	78.9	98.5	8	Parotid & submandibular gland, minor salivary glands
Wu 2019^17^—multicentric	694 (1560)	18.3	8.9	37.5	2.9	40.7	100.0	98.3	12	Parotid & submandibular & sublingual gland, minor salivary glands, salivary gland NOS
Park 2019^4^—unicentric	413 (413)	19.5	6.9	0.0	2.4	26.2	83.3	100.0	6	Parotid gland
Behaeghe 2020^33^—unicentric	235 (359)	13.8	14.2	30	6.3	20.8	60.0	100.0	6	NA^c^
Lee 2020^21^—unicentric	421 (1384)	10.0	17.5	29.5	0.5	17.1	83.3	100.0	10	Parotid & submandibular gland, minor salivary glands, salivary gland NOS^d^
Lubin 2020^25^—unicentric	373 (976)	20.7	30.0	45.8	3.3	50.7	100.0	100.0	10	NA
Mazzola 2020^22^—multicentric	503 (503)	19.5	14.3	17.6	3.6	24.6	66.7	96.8	7	Parotid & submandibular gland^e^
Reerds 2021^14^—multicentric	9672 (9672)	12.5	10.3	29.0	2.3	28.6	83.0	99.3	11	Parotid gland^f^
Aksoy Altinboga 2021^26^—unicentric	198 (578)	13.0	7.0	23.0	1.1	53.3	100.0	100.0	10	Parotid & submandibular gland, minor salivary glands
Higuchi 2022^27^—multicentric	1608 (1608)	13.4	9.1	24.9	1.8	37.0	89.7	99.3	13	Parotid & submandibular gland, other
Lui 2022^34^—unicentric	372 (819)	37.5	31.3	52.2	3.8	41.3	92.3	99.0	11	NA
Total	17,413 (22834)	15.7	12.3	26.9	2.9	35.7	84.8	98.5		
Current study—unicentric	753 (2156)	26.7	5.7	34.0	1.1	21.8	92.0	99.2	10	Parotid & submandibular gland, minor salivary glands, salivary gland NOS
MSRSGC classification		25.0	10.0	20.0	<5	35.0	60.0	90.0		

^a^ Number of FNAC with histopathological follow‐up and in brackets the total amount of FNAC.

^b^ Exclusion of intraglandular metastasis.

^c^ Only samples processed as cell blocks were included.

^d^ Exclusion of intraparotid lymph node cases.

^e^ Exclusion of FNAC performed by external physicians.

^f^ Exclusion of lymphoma.

## 4 DISCUSSION

To our knowledge, our analysis of 2156 FNA of SG lesions with available histologic correlation in 753 cases is the largest single‐center study in the field. We could show that FNAC and the categorization based on the MSRSGC are useful in clinical application. The sensitivity and specificity for predicting malignancy were 94.6% and 99.3%, respectively, which affirms the high accuracy of SG FNAC. The ROM across the different categories is within the range of values provided by MSRSGC, but with some variations. Discrepancies were noted primarily in categories I, III, and IVB. These are the categories where the final diagnosis is unclear and further investigation is required. The results of our study show better values in the benign categories II and IVA, as well as in the malignant categories V and VI, than provided by MSRSGC (see Table 2). Comparison with other studies revealed variations in the distribution within the different categories, especially in categories I, II, III, and IVB (Table 3).

### 4.1 Non‐diagnostic

According to Faquin et al^13^ non‐diagnostic FNA should not exceed 10% to fulfill quality requirements. In our cohort, a total of 8% of FNA and 6% of cases with available histology were non‐diagnostic. Significant differences were detected in non‐diagnostic rates between FNA performed by non‐cytopathologists (29%) and cytopathologists at the UHZ (5%). Since no rapid on‐site evaluation was conducted in either external or internal FNA, this can be excluded as cause of the difference. A possible explanation is the varying level of experience. This is also reflected by the fact that cell‐poor or artifact‐ridden smears were the main findings in non‐diagnostic FNA (62.2%). Especially in pleomorphic adenomas (which represented 28.9% in the definite histologic workup in this group), the diagnostic yield can be low in unexperienced hands. In 13.4% cytology showed only normal tissue without lesional components, indicating a sampling error, maybe due to inadequate clinical information or incorrect radiological correlation. The size may also affect the difficulty of FNA. Accordingly, we found that the average diameter was slightly smaller than the average size in other categories, not including cystic lesions. Altogether, this suggests that FNA of SG lesions should be performed in experienced centers to keep the number of non‐diagnostic results low. Another reason for non‐diagnostic aspirates might be the low cellularity of the lesions themselves. Typical examples are hemangiomas, lipomas, and fibrosing lesions such as scars or atrophic tissue. However, some SG tumors are also rich in fibrous stroma, which can lead to non‐diagnostic aspirates.

According to MSRSGC, FNA with exclusive non‐mucinous cyst content should be classified as non‐diagnostic,^9^, ^13^ since the spectrum of cystic‐transformed lesions is extremely broad. In our daily practice, non‐mucinous cystic lesions with complete regression after FNA matching ultrasound and clinical appearance are classified as benign cysts and placed in category II. This approach leads to a low percentage of non‐diagnostic FNA. Several previous studies report a higher rate (>10%) of non‐diagnostic cases when the MSRSGC criteria are strictly applied.^6^, ^14^, ^15^, ^21^, ^23^, ^24^, ^27^ In our study, unclear cystic lesions represented 24.4% of category I cases, which were finally diagnosed as cystic‐transformed SG tumors in histology—most commonly Warthin tumors, but also other tumors with unusual pronounced cystic degeneration (e.g., pleomorphic adenoma, oncocytoma, cystadenoma, and mucoepidermoid carcinoma).

Our ROM in category I was slightly higher (26.7%) compared to original MSRSGC (25%) and previous studies (on average 15.7%, Table 3). This can be partly explained by the fact that we placed non‐mucinous cysts—if clinically and radiologically appropriate for a cyst—in category II. Although this kept the rate of non‐diagnostic FNA low, it led to an increase in ROM. If we had placed non‐mucinous cysts strictly in category I, ROM in this category would have decreased to 20.0% and increased to 7.3% for category II. Additionally, only a few category I cases were resected (26.3%). This represents a selection bias for ROM and RON, which are calculated based only on cases with available histology. In reality, ROM and RON are therefore likely to be lower. A similar effect was observed in the studies of Lubin et al^25^ and Mazzola et al.^22^ It is also possible that suspicious lesions underwent direct surgery without repeat FNA, whereas inconspicuous lesions continued to be observed and disappeared during follow‐up. Another relevant finding was the higher percentage of non‐diagnostic FNA performed by non‐cytopathologists (29%) in comparison with cytopathologists (5%), resulting in different ROM values (33.4% versus 23.3%). Finally, a second FNA was performed in 23% of initially non‐diagnostic cases, of which 86.5% were eventually diagnostic. This approach also supports the recommendations by MSRSGC to correlate lesions clinically and radiologically and to repeat FNA in doubtful cases.^9^, ^13^

### 4.2 Non‐neoplastic

This category included mainly reactive or inflammatory processes, such as abscesses, chronic or granulomatous inflammation, accounting for 32.9%. In our study, 25.7% of cases were cystic non‐neoplastic lesions with a broad spectrum in the resected specimens like epidermoid cysts, ranulae, mucoceles, thyroglossal duct cysts, lymphoepithelial cysts, branchiogenic cysts, and others, but also 17.1% cystic‐transformed benign tumors. 10% of cases were reactive lymph nodes.

Category II quantitatively accounted for the second largest category (32%) of all performed SG FNA. However, only 10.1% of these lesions were resected, so ROM is probably overestimated. Nevertheless, at 5.7%, our ROM was well below the 10% ROM rate reported by the MSRSGC and below the ROM rate of most previously conducted studies as well (Table 3). Overall, false‐negative results were limited to 4 out of 70 cases (5.7%), half of which were lymphomas (misinterpreted as reactive lymph nodes). This reflects the results of the studies by Rossi et al,^9^ Viswanathan et al,^15^ and Song et al,^18^ which also found B‐cell lymphomas as a main reason of false‐negative diagnoses. In general, diagnosis and subclassification of low‐grade lymphoma are well known limitations of FNAC.^28^ Moreover, the FNA diagnosis of “reactive or normal intraparotid lymph node” (*n* = 13) was only accurate in 53.8% of subsequent excisions. Apart from the two already mentioned low‐grade lymphomas, two lymph node metastases (one sclerotic variant of a mucoepidermoid carcinoma and one melanoma) were found in the resections—both small and in the marginal area of a lymph node, indicating a sampling error. Furthermore, two Warthin tumors were misinterpreted once as a lymph node and once as a lymphoepithelial cyst. Although the abovementioned cases were cytologically classified as non‐neoplastic, they were nevertheless subsequently resected due to persistent clinical and/or radiological suspicion for malignancy.

A striking feature in our study was the high RON of 24.3% in this category. In addition to the aforementioned malignant neoplasms, this category also contained benign neoplasms like Warthin tumors (9/70) and cystadenomas (2/70), which were cytologically interpreted predominantly as cysts and occasionally as inflammatory lesions or lymph nodes. Subtotally cystic regressed neoplasms are inadequately identified in this regard. Previously performed studies that provided data for RON, reported similar or even higher RON rates with 21.4%^17^ and 47.6%^21^ but with a smaller number of cases (*n* = 14 and *n* = 21).

### 4.3 Atypia of undetermined significance (AUS)

According to the MSRSGC, this category should not exceed 10%.^9^, ^13^ Our rates were 5% overall and 6.6% for the cases with correlating histopathological diagnosis, which showed a wide spectrum of benign and malignant processes: Warthin tumor (12/50), lymphoma (7/50), pleomorphic adenoma (5/50), cysts (5/50), reactive lymph nodes (4/50), inflammatory changes (4/50), and squamous cell carcinomas (4/50). One typical cytological finding in this diagnostically inconclusive category is a monotonous population of small lymphocytes that may indicate low‐grade lymphoma but may also be present in Warthin tumors, intraglandular lymph node metastasis, or inflammatory processes.^5^ ROM in this category was 34% and thus clearly higher than the provided 20% of the MSRSGC. However, a comparison with previously conducted studies showed that most ROM in the AUS category were above the proposed 20% of MSRSGC (in average 26.9%, Table 3). Finally, a second FNA was performed in 13% of initially as AUS interpreted cases in our study, of which 71.4% were eventually diagnostic. This approach and the relatively high ROM support the recommendations by MSRSGC to repeat FNA or to go for surgery.

### 4.4 Benign neoplasm

The numerically largest category was category IVA (48.7% of FNAC with histopathological follow‐up). Half of the excised lesions in this category were pleomorphic adenomas and 41.4% Warthin tumors. Accordingly, RON was high (99.2%), whereas ROM was very low (1.1%), adhering to the established ROM of <5% by MSRSGC. Overall, 74.5% of pleomorphic adenomas and 80.9% of Warthin tumors could be accurately diagnosed in our study. The high accuracy can be explained by straight forward diagnostic features. Nevertheless, four false‐negative results were found, one being a lymphoma and one a mucoepidermoid carcinoma, both misinterpreted as Warthin tumor. The other two false‐negative diagnoses were an adenoid cystic carcinoma and an epithelial‐myoepithelial carcinoma, which were misinterpreted as pleomorphic adenomas.

A second FNA was performed in 2% of category IVA cases. In most cases, it was performed with an average interval of 2.4 years in Warthin tumors (60%) that were repunctured due to an increase in size during follow‐up.

### 4.5 SUMP

This category includes neoplasms for which no specific subtype can be determined cytologically, and therefore, no classification into benign and malignant can be made.^9^ Smears are mainly highly cellular without striking nuclear atypia, resulting in morphologic overlap between benign tumors and low‐grade carcinomas. A typical benign example is the cellular variant of pleomorphic adenoma, which was indeed the most common histology in this category (*n* = 45, 57.7%), followed by other benign tumors, such as lymphocyte poor Warthin tumor and basal cell adenoma (*n* = 6, 7.7% each). However, also malignant tumors were diagnosed including three adenoid cystic carcinomas, three epithelial‐myoepithelial carcinomas, two myoepithelial carcinomas, two salivary duct carcinomas, two squamous cell carcinomas and others. The ROM value was 21.8%, lower than the MSRSGC data (35%) and lower than most previous studies. Liu et al^29^ and Mazzola et al^22^ showed similar low ROM for the SUMP category. They considered a possible selection bias because of their low number of cases in this category: Liu et al^29^ with 54 cases, Mazzola et al^22^ with 61 cases (our study with 78 cases).

In this category, MSRSGC recommends conservative surgery as further clinical management to clarify the diagnosis and behavior of the tumor. From a surgical point of view, a definite diagnosis would significantly influence planning and extent of resection. Although category IVB accounted for only 5% in our study, reducing the number of cases in this category should be the goal.

In the last years, an increasing number of SG neoplasms have been characterized on a molecular level.^30^, ^31^ Therefore, ancillary tests such as immunohistochemistry, fluorescence in situ hybridization or sequencing‐based methods will probably further significantly increase the diagnostic accuracy of preoperative cytologic specimens and reduce the number of cases of SUMP, but also in the uncertain categories III and V.^30^, ^31^ Recently, the SG neoplasm specific next generation sequencing panel (“SalvGlandDx”) has been developed at the UHZ. It offers the advantage of simultaneous detection of aberrant expression, gene mutations, and fusions by RNA extraction. This NGS‐based panel can also be applied to cell block material from FNA and is suitable as a useful all‐in‐one tool for diagnosis and more accurate classification of SG neoplasms.^32^ A study investigating this aspect is currently underway at the UHZ.

### 4.6 Suspicious for malignancy

This category contained the fewest cases (3.3% with histopathological follow‐up). ROM and RON were both high (92%). The ROM provided by MSRSGC is 60%. In comparison with previous studies, our value lies in the upper range, but all studies showed values above 60% with an average of 84.8% (Table 3).

Half of the definite histopathological diagnoses were different types of lymphomas, which were also correctly classified in the previous FNAC. There were two false‐positive diagnoses: In one case, cytology was assessed as “cells with atypia, suspicious for malignancy”, whereas histology revealed a benign cyst with excessive degenerative atypia. The other was diagnosed as “suspicious for a malignant neuroendocrine neoplasm” based on immunohistochemical examination of the cell block. Final histological workup showed a central fibrotic area in otherwise normal parenchyma without evidence of malignancy. The interval between FNA and surgery was approximately 1 month. A potential sampling error was discussed, but the patient was lost for follow‐up.

### 4.7 Malignant

15.7% of all FNAC with histopathological follow‐up were classified malignant. ROM and RON were high with 99.2% and 100%, respectively, and thus higher than the provided data by MSRSGC (90%). All reviewed previous studies in the literature reported ROM > 90%. In our study, the most frequently diagnosed malignant histopathological subtypes in this category were squamous cell carcinoma (17.8%), lymphoma (16.9%), and melanoma (14.4%). The most frequently diagnosed malignant tumors originating from the SG were acinic cell carcinoma (9.3%) and salivary duct carcinoma (8.5%). We detected one false‐positive case: A Warthin tumor with squamous metaplasia was misdiagnosed as a lymph node metastasis of a squamous cell carcinoma in FNAC.

All histopathologic entities in every MSRSGC category are provided in Table S2.

## 5 CONCLUSION

We present our experience with the MSRSGC at the UHZ, which to our knowledge is the largest single‐center study in this field. The high sensitivity of 94.6% and specificity of 99.3% to distinguish between non‐neoplastic and benign neoplastic versus malignant neoplastic lesions affirm the high accuracy of SG FNAC. Our study provides additional evidence that the MSRSGC categorization is valuable, and it confirms its effectiveness and validity. The calculated ROM largely met the thresholds of MSRSGC and previously conducted studies. One exception was category I with a higher ROM than expected. This is due to our practice of moving the contents of non‐mucinous cysts to category II when the clinical context is appropriate. In addition, we demonstrated that cytopathologists had a significantly lower rate of non‐diagnostic FNA, suggesting that cytologic workup of SG lesions should be performed in centers with appropriate experience. However, despite the advantages of preoperative FNAC, this procedure has its limitations. This is mainly also due to the complexity of the tumors themselves and their morphological overlap. However, we expect that the increased use of ancillary tests will further improve diagnostic accuracy in the near future.

## AUTHOR CONTRIBUTIONS

**Giulia Tochtermann:** Conceptualization (supporting); data curation (equal); formal analysis (lead); methodology (equal); project administration (equal); visualization (lead); writing – original draft (lead). **Miriam Nowack:** Conceptualization (equal); data curation (equal); formal analysis (supporting); methodology (equal); validation (equal); writing – original draft (supporting); writing – review and editing (equal). **Cristina Hagen:** Data curation (supporting); investigation (lead); writing – review and editing (equal). **Niels J. Rupp:** Writing – review and editing (equal). **Kristian Ikenberg:** Writing – review and editing (equal). **Martina A. Broglie:** Writing – review and editing (equal). **Francesca Saro:** Writing – review and editing (equal). **Daniela Lenggenhager:** Writing – review and editing (equal). **Peter K. Bode:** Conceptualization (equal); data curation (equal); formal analysis (equal); methodology (equal); supervision (lead); validation (equal); writing – original draft (supporting); writing – review and editing (lead).

## FUNDING INFORMATION

The authors received no specific funding for this work.

## CONFLICT OF INTEREST STATEMENT

None to declare.

## ETHICS STATEMENT

This study was conducted in accordance with the Declaration of Helsinki and its amendments. According to swissethics, quality assurance projects do not require ethical approval. General consent was obtained before FNA procedure.

## Supporting information


Table S1.

Table S2.

## Data Availability

The datasets used and analyzed during the current study are available from the corresponding author on reasonable request.
