# Supplementary material for: The Milan system for reporting salivary gland cytopathology—A single‐center study of 2156 cases
Source: Cancer Med. 2023 Apr 16;12(11):12198–207. doi: 10.1002/cam4.5914 (PMC10278457; doi:10.1002/cam4.5914)
Supplement: Supplementary file 1 — Table S1. [file CAM4-12-12198-s002.docx]

| **Benign and Malignant Salivary Gland Neoplasms (No.)** | | **MSRSGC Category (No.)** | | | | | | |
| --- | --- | --- | --- | --- | --- | --- | --- | --- |
|  |  | **I** | **II** | **III** | **IVA** | **IVB** | **V** | **VI** |
| **BENIGN** | Pleomorphic adenoma (248) | 5.2% (13) | 0.8% (2) | 2% (5) | **74.2% (183)** | 18.1% (45) |  |  |
|  | Warthin tumor (188) | 4.1% (8) | 4.8% (9) | 6.4% (12) | **80.9% (152)** | 3.2% (6) |  | 0.5% (1) |
|  | Lipoma (14) | 14.3% (2) |  |  | **85.7% (12)** |  |  |  |
|  | Basal cell adenoma (13) | 7.7% (1) |  |  | **46.2% (6)** | 46.2% (6) |  |  |
|  | Oncocytoma (8) | 25% (2) |  | 25% (2) | **37.5% (3)** | 12.5% (1) |  |  |
| **MALIGNANT** | Lymphoma (43) | 2.3% (1) | 4.7% (2) | 16.3% (7) | 2.3% (1) |  | 27.9% (12) | **46.5% (20)** |
|  | Metastasis of squamous cell carcinoma (29) | 7.1% (2) |  | 14.3% (4) |  | 7.1% (2) |  | **71.4% (21)** |
|  | Melanoma (19) | 5.26% (1) | 5.26% (1) |  |  |  |  | **89.5% (17)** |
|  | Mucoepidermoid carcinoma (14) | 21.4% (3) | 7.1% (1) | 14.3% (2) | 7.1% (1) | 7.1% (1) | 14.3% (2) | **28.6% (4)** |
|  | Salivary duct carcinoma (14) | 7.1% (1) |  |  |  | 14.3% (2) | 7.1% (1) | **71.4% (10)** |
|  | Acinic cell carcinoma (13) | 7.7% (1) |  |  |  |  | 7.7% (1) | **84.6% (11)** |
|  | Adenoid cystic carcinoma (13) | 7.7% (1) |  | 15.4% (2) | 7.7% (1) | 23.1% (3) | 15.4% (2) | **28.6% (4)** |
|  | Carcinoma ex pleomorphic adenoma (7) |  |  |  |  | 14.3% (1) | 14.3% (1) | **71.4% (5)** |
|  | Myoepithelial carcinoma (5) |  |  |  |  | 40% (2) | 20% (1) | **40% (2)** |
|  | Epithelial-myoepithelial carcinoma (5) |  |  |  | 20% (1) | 60% (3) |  | **20% (1)** |
|  | Basal cell adenocarcinoma (5) |  |  |  |  | 20% (1) | 20% (1) | **60% (3)** |

**Supporting Information 1.** Distribution of the Most Frequent Benign and Malignant Neoplasms within the MSRSGC Category

Abbreviations: MSRSGC, Milan System for Reporting Salivary Gland Cytopathology
