# Supplementary material for: The Milan system for reporting salivary gland cytopathology—A single‐center study of 2156 cases
Source: Cancer Med. 2023 Apr 16;12(11):12198–207. doi: 10.1002/cam4.5914 (PMC10278457; doi:10.1002/cam4.5914)
Supplement: Supplementary file 2 — Table S2. [file CAM4-12-12198-s001.docx]

| **Entities in**  **Category I (No.)** | **Entities in**  **Category II (No.)** | **Entities in**  **Category III (No.)** | **Entities in**  **Category IVA (No.)** | **Entities in**  **Category IVB (No.)** | **Entities in**  **Category V (No.)** | **Entities in**  **Category VI (No.)** |
| --- | --- | --- | --- | --- | --- | --- |
| **Non-neoplastic** | **Non-neoplastic** | **Non-neoplastic** | **Non-neoplastic** | **Non-neoplastic** | **Non-neoplastic** | **Non-neoplastic** |
| Normal parenchyma (1) | Inflammation, chronic/acute (20) | Cyst (5) | Cyst (2) | Cyst (1) | Cyst (1) | - |
| Atrophy (1) | Cyst (18) | Lymph node (4) | Metaplasia (1) | **Neoplastic, benign** | Normal parenchyma (1) | **Neoplastic, benign** |
| Scar (1) | Lymph node (7) | Inflammation, chronic/acute (2) | **Neoplastic, benign** | PA (45) | **Neoplastic, benign** | WT (1) |
| **Neoplastic, benign** | Inflammation, granulomatous (3) | Inflammation, granulomatous (2) | PA (183) | WT (6) | - | **Neoplastic, malignant** |
| PA (13) | Scar (2) | Normal parenchyma (1) | WT (152) | BCA (6) | **Neoplastic, malignant** | SCC (21) |
| WT (8) | Atrophy (1) | **Neoplastic, benign** | Lipoma (12) | Myoepithelioma (1) | Lymphoma (12) | Lymphoma (20) |
| Lipoma (2) | Ganglion (1) | WT (12) | BCA (6) | Oncocytoma (1) | MEC (2) | Melanoma (17) |
| Oncocytoma (2) | Vascular malformation (1) | PA (5) | Oncocytoma (3) | Nodular fasciitis (1) | AdCC (2) | ACC (11) |
| Haemangioma (2) | **Neoplastic, benign** | Oncocytoma (2) | Cystadenoma (1) | **Neoplastic, malignant** | SDC (1) | SDC (10) |
| BCA (1) | WT (9) | **Neoplastic, malignant** | Neurofibroma (1) | EMC (3) | ACC (1) | Adeno-Ca NOS (8) |
| Myoepithelioma (1) | PA (2) | Lymphoma (7) | Paraganglioma (1) | AdCC (3) | MC (1) | Ca-ex-PA (5) |
| Cystadenoma, oncocytic (1) | Cystadenoma (2) | SCC (4) | Benign fibrous histiocytoma (1) | SDC (2) | Ca metastasis (1) | MEC (4) |
| **Neoplastic, malignant** | **Neoplastic, malignant** | MEC (2) | **Neoplastic, malignant** | SCC (2) | Adeno-Ca NOS (1) | AdCC (4) |
| MEC (3) | NHL (2) | AdCC (2) | MEC (1) | MC (2) | Ca-ex-PA (1) | Ca metastasis (4) |
| SCC (2) | MEC (1) | Carcinosarcoma ex PA (1) | EMC (1) | MEC (1) | BCAdc (1) | BCAdc (3) |
| SDC (1) | Melanoma metastasis (1) | Ca metastasis (1) | AdCC (1) | Ca metastasis (1) |  | MC (2) |
| AdCC (1) |  |  | Lymphoma (1) | Intraductal Ca (1) |  | Merkel cell Ca (2) |
| ACC (1) |  |  |  | Ca-ex-PA (1) |  | SC (2) |
| Melanoma (1) |  |  |  | BCAdc (1) |  | EMC (1) |
| Adeno-Ca NOS (1) |  |  |  |  |  | LEC (1) |
| Neuroendocrine Ca (1) |  |  |  |  |  | Melanoma metastasis (1) |
| NHL (1) |  |  |  |  |  | Oflactory neuroblastoma (1) |

**Supporting Information 2.** Categorization of Histopathological Diagnoses within the MSRSGC Categories

Abbreviations: PA, Pleomorphic Adenoma; WT, Warthin Tumor; BCA, Basal Cell Adenoma; MEC, Mucoepidermoid Carcinoma; SCC, Squamous Cell Carcinoma; SDC, Salivary Duct Carcinoma; AdCC, Adenoid Cystic Carcinoma; ACC, Acinic Cell Carcinoma; Ca, Carcinoma; NOS, Not Otherwise Specified; NHL, Non Hodgkin Lymphoma; EMC, Epithelial-Myoepithelial Carcinoma; MC, Myoepithelial Carcinoma; BCAdc, Basal Cell Adenocarcinoma; SC, Secretory Carcinoma; LEC, Lymphoepithelial Carcinoma
